# Analyses of histological and transcriptome differences in the skin of short-hair and long-hair rabbits

**DOI:** 10.1186/s12864-019-5503-x

**Published:** 2019-02-15

**Authors:** Haisheng Ding, Huiling Zhao, Guanglong Cheng, Yongxin Yang, Xiaofei Wang, Xiaowei Zhao, Yunxia Qi, Dongwei Huang

**Affiliations:** Anhui Key Laboratory of Livestock and Poultry Product Safety Engineering, Institute of Animal Husbandry and Veterinary Medicine, Anhui Academy of Agricultural Sciences, Hefei, 230031 Anhui People’s Republic of China

**Keywords:** Skin, Hair fibre length, Histological analysis, Gene expression, RNA sequencing, Rabbit

## Abstract

**Background:**

Hair fibre length is an important economic trait of rabbits in fur production. However, molecular mechanisms regulating rabbit hair growth have remained elusive.

**Results:**

Here we aimed to characterise the skin traits and gene expression profiles of short-hair and long-hair rabbits by histological and transcriptome analyses. Haematoxylin-eosin staining was performed to observe the histological structure of the skin of short-hair and long-hair rabbits. Compared to that in short-hair rabbits, a significantly longer anagen phase was observed in long-hair rabbits. In addition, by RNA sequencing, we identified 951 genes that were expressed at significantly different levels in the skin of short-hair and long-hair rabbits. Nine significantly differentially expressed genes were validated by quantitative real-time polymerase chain reaction. A gene ontology analysis revealed that epidermis development, hair follicle development, and lipid metabolic process were significantly enriched. Further, we identified potential functional genes regulating follicle development, lipid metabolic, and apoptosis as well as important pathways including extracellular matrix-receptor interaction and basal cell carcinoma pathway.

**Conclusions:**

The present study provides transcriptome evidence for the differences in hair growth between short-hair and long-hair rabbits and reveals that lipid metabolism and apoptosis might constitute major factors contributing to hair length.

**Electronic supplementary material:**

The online version of this article (10.1186/s12864-019-5503-x) contains supplementary material, which is available to authorized users.

## Background

Rabbits are small mammals providing not only meat, but also hair. Hair is produced from follicles as skin appendages unique to mammals that are characterised by periodic regrowth [1, 2]. Rabbit hair is one of the most favourite natural fibres used in textile industries. The key traits contributing to the economic value of rabbit hairs include fibre diameter, density, and length, which are determined by both genetics and the environment [3–6]. The mechanisms regulating hair traits are complicated. Understanding the genetic principles of hair traits could help to elucidate the mechanism of hair development, thus, promoting rabbit breeding.

Hair fibre length is a critical economic trait in wool production from Angora rabbits, as it is closely associated with wool quality and yield. In addition, hair length influences textile quality, and also determines the system of manufacture. Different types of hairs in rabbits possess different growth rates, which vary with breed, nutrition, and season [4]. For Angora rabbits in the same environment conditions, gender, body site, and the month age after plucking are closely related to growth rates of rabbit hairs [4]. Hair follicles are the bases of hair growth. An intact hair coat is maintained by lifelong cycling of the hair follicles through periodic stages of growth (anagen), regression (catagen), and relative quiescence (telogen) [2, 7–9]. During the anagen phase, hair follicles grow rapidly by cell proliferation and reach their longest length. After regression by apoptosis in the catagen phase, they enter the telogen phase [10, 11]. Apoptosis is a critical event in the regulation of the hair cycle as anagen hairs normally grow for 4–7 years in humans before the cycle enters into the resting phases of catagen and telogen [12, 13]. During the process of hair follicles development, Wnt signalling plays a crucial role in hair follicle morphogenesis and regeneration [14, 15]. In addition, some signalling molecules, such as bone morphogenetic protein (BMP), sonic hedgehog (SHH), fibroblast growth factor (FGF), and transforming growth factor (TGF)-β, are involved in regulating the time of entry into the anagen phase of the hair follicle cycle [16–18]. Hair growth is also regulated by insulin-like growth factor 1 (IGF-1) affecting human follicular proliferation, hair growth cycle, and follicular differentiation [19]. FGF5, a member of the FGF family, is the leading candidate for hair length variation, and it is considered a regulator determining the duration of active hair growth (anagen) and the time of hair follicle regression (catagen) [20–22]. Recent studies have showed that VEGFR-2 and transcription factor NF-κB are involved in hair cycle regulation and NF-κB is indispensable for hair follicle stem cell activation, maintenance, and/or growth in mice [23, 24].

With the rapid development of molecular biological techniques, especially next-generation sequencing, large-scale gene expression detection has become possible. Rabbit genome sequencing has been used to study the polygenic basis of phenotypic variation during rabbit domestication [25] and differential gene expression in parthenogenetic and normal embryos cultured under the same conditions [26]. The landscape of long non-coding RNAs has also been analysed in *Trichophyton mentagrophytes*-induced dermatophytosis lesional skin and normal skin of rabbit by RNA sequencing (RNA-Seq) [27]. De novo transcriptome sequencing of Rex rabbit has been performed, and some novel genes have been found to play important roles during skin growth and development [28]. In the present study, to gain a better understanding of the growth rhythm of rabbit hair and molecular mechanisms regulating hair length in rabbits, a histological comparison of hair follicle phenotypes and transcriptome analysis were performed between short-hair and long-hair rabbits. Our results elucidate the molecular mechanism of hair growth and provide valuable information for future studies.

## Results

### Histological analysis of skin and follicle morphology of short-hair and long-hair rabbits

**Fig. 1:**
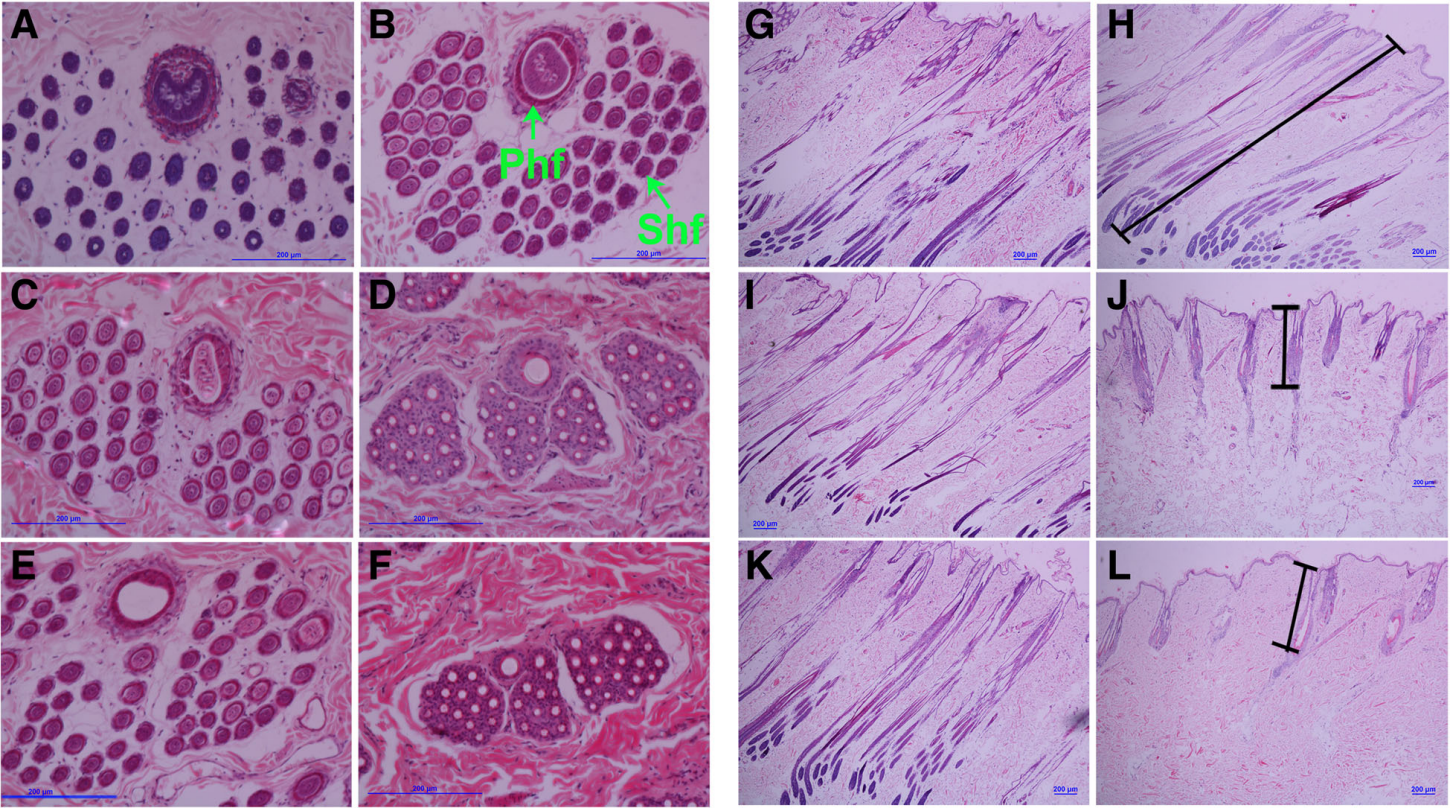
The histological observation of skin tissue after plucking out hairs from long-hair and short-hair rabbits. **a** Transverse section of long-hair at the fourth week. **b** Transverse section of short-hair rabbit at the fourth week. **c** Transverse section of long-hair at the sixth week. **d** Transverse section of short-hair rabbit at the sixth week. **e** Transverse section of long-hair at the eighth week. **f** Transverse section of short-hair rabbit at the eighth week. **g** Longitudinal section of long-hair at the fourth week. **h** Longitudinal section of short-hair rabbit at the fourth week. **i** Longitudinal section of long-hair at the sixth week. **j** Longitudinal section of short-hair rabbit at the sixth week. **k** Longitudinal section of long-hair at the eighth week. **l** Longitudinal section of short-hair rabbit at the eighth week. Phf-Primary hair follicles, Shf-Secondary hair follicles, Bars = 200 μm

Complete hair follicle structure was observed in short-hair and long-hair rabbits at the fourth week of hair growth (Fig. 1a, b, g, h), which indicated that the hair follicles were in the anagen phase in short-hair and long-hair rabbits. Compared with that at the fourth week, no obvious changes were observed in long-hair rabbits at the sixth week (Fig. 1c, i). However, the hair follicles of short-hair rabbits appeared shrunk at the sixth week (Fig. 1d), and the area of primary and secondary hair follicles and thickness of inner root sheaths obviously decreased when compared with those at the fourth week (Fig. 1d, j). The hair follicle structure remained intact at the eighth week in long-hair rabbits (Fig. 1e, k), whereas, the hair follicles of short-hair rabbits significantly shrunk and finger-like papillae atrophied up to the arrectores pilorum, which indicated that the hair follicles of short-hair rabbits at the eighth week had entered the telogen phase, resulting in the cessation of hair growth (Fig. 1f, l). However, the hair follicles of long-hair rabbits were still in the anagen phase until at the eighth week, leading to continuous growth of hair.

### Transcriptome of rabbit skin by RNA sequencing

**Fig. 2:**
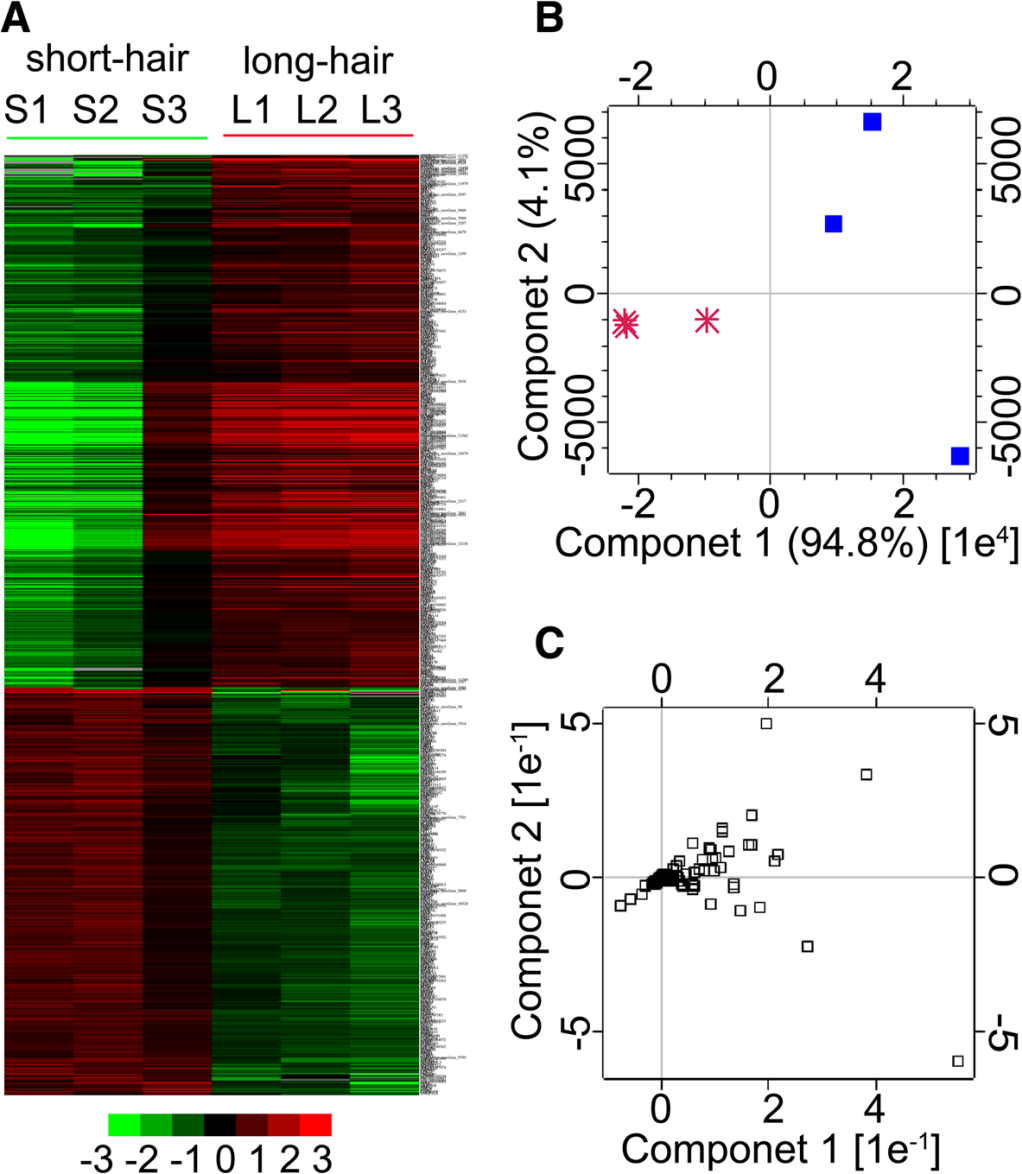
A heatmap and Principal Component Analysis of the DEGs between short-hair and long-hair rabbit. **a** Cluster analysis of DEGs. The red colour indicates increased expression and the green colour indicates decreased expression. S represents short-hair rabbits; L represents long-hair rabbits. **b** PCA of DEGs between short-hair and long-hair rabbit. The two rabbit types were clearly separated along the direction of component 1. The star represents long-hair rabbit; the solid square represents short-hair rabbit. **c** PCA of DEGs. The hollow box represents DEGs. The genes crucial for hair length regulation were identified by PCA

Six cDNA libraries of the long-hair rabbit groups (L1, L2, and L3) and short-hair rabbit groups (S1, S2, and S3) were constructed. After removing low-quality reads and adaptor sequences, more than 36 M clean reads were obtained from each sample, and Q30 was ≥91.74% (Table 1; Additional file 1: Figure S1). The map rates of unique reads from the sequenced skin RNA of long-hair and short-hair rabbits were > 60.45% (Table 2). After the standard analysis, 16,931 genes were detected from the six samples. Interestingly, the number of genes found among all the samples was similar with respect to the FPKM value (Additional file 2: Figure S2). The largest proportion of genes exhibited low (0 < FPKM < 10) and moderate (10 < FPKM < 100) expression, and a small fraction of the genes were expressed at high levels (FPKM > 100), further confirming the advantage of detecting low-abundance genes by high-throughput sequencing. In addition, 951 DEGs (FDR < 0.01, |log2Ratio| ≥ 1) were filtered between the groups comprising 539 genes expressed highly in the skin of long-hair rabbits and 412 genes expressed highly in the skin of short-hair rabbits (Fig. 2a; Additional file 3: Figure S3). A number of genes that were identified as being more highly expressed in long-hair rabbits were from the keratin (KRT) families, including the 10 genes (*KRT23*, *KRT25*, *KRT26*, *KRT28*, *KRT34*, *KRT38*, *KRT39*, *KRT40*, *KRT7*, and *KRT84*) (Table 3), which suggested that the 10 genes are important candidates regulating hair length in rabbits. In addition, a principal component analysis (PCA) was performed on DEGs, which showed that the short-hair and long-hair rabbits were clearly differentiated in the direction of the component 1 (Fig. 2b). Moreover, PCA identified several genes functioning in hair growth regulation, such as *KRT25*, *S100A3*, *COL3α1*, *COL1α2*, *LOC100340949*, *LOC100338342*, *LOC100359226* (Fig. 2c).

**Table 1:** Summary of RNA-seq data for each sample

Samples	Clean reads	Clean bases	≥ Q30 (%)
L1	47,547,114	13,997,256,514	91.76%
L2	36,468,426	10,495,999,462	92.39%
L3	57,393,736	16,932,383,874	92.02%
S1	46,632,332	13,639,996,064	93.44%
S2	46,105,321	13,567,869,214	92.01%
S3	49,671,512	14,615,589,684	91.74%

**Table 2:** Mapping statistics for each sample

Samples	Clean reads	Mapped reads	Unique mapped reads	Multiple mapped reads
L1	95,094,228	62,621,046 (65.85%)	59,482,256 (62.55%)	3,138,790 (3.30%)
L2	72,936,852	46,536,580 (63.80%)	44,088,800 (60.45%)	2,447,780 (3.36%)
L3	114,787,472	77,804,317 (67.78%)	73,059,536 (63.65%)	4,744,781 (4.13%)
S1	93,264,664	69,770,638 (74.81%)	67,443,108 (72.31%)	2,327,530 (2.50%)
S2	92,210,642	60,012,231 (65.08%)	57,464,532 (62.32%)	2,547,699 (2.76%)
S3	99,343,024	65,785,338 (66.22%)	62,960,030 (63.38%)	2,825,308 (2.84%)

Clean reads are single-end Reads

**Table 3:** KRT genes from KRT family differentially expressed in the skin of short-hair and long-hair rabbits. The keratin filament (GO:0045095) and intermediate filament (GO:0005882) in the cellular component category were enriched by KRT7 and KRT34, respectively. The epithelial cell differentiation (GO:0030855) in the biological process category was enriched by KRT40

Gene symbol	Type	log2^FoldChange^	FDR	GO term
KRT7	II	2.390336	4.68E-05	keratin filament (GO:0045095)
KRT23	I	1.250867	0.005003	NG
KRT25	I	2.695538	4.30E-05	NG
KRT26	I	3.158961	2.08E-05	NG
KRT28	I	2.377374	0.002598	NG
KRT34	I	3.512258	8.31E-10	intermediate filament (GO:0005882)
KRT38	I	3.924441	3.98E-07	NG
KRT39	I	2.660676	1.92E-06	NG
KRT40	I	2.310793	4.14E-05	epithelial cell differentiation (GO:0030855)
KRT84	II	3.162653	1.44E-13	NG

### Verification of DEGs with quantitative real-time PCR

**Fig. 3:**
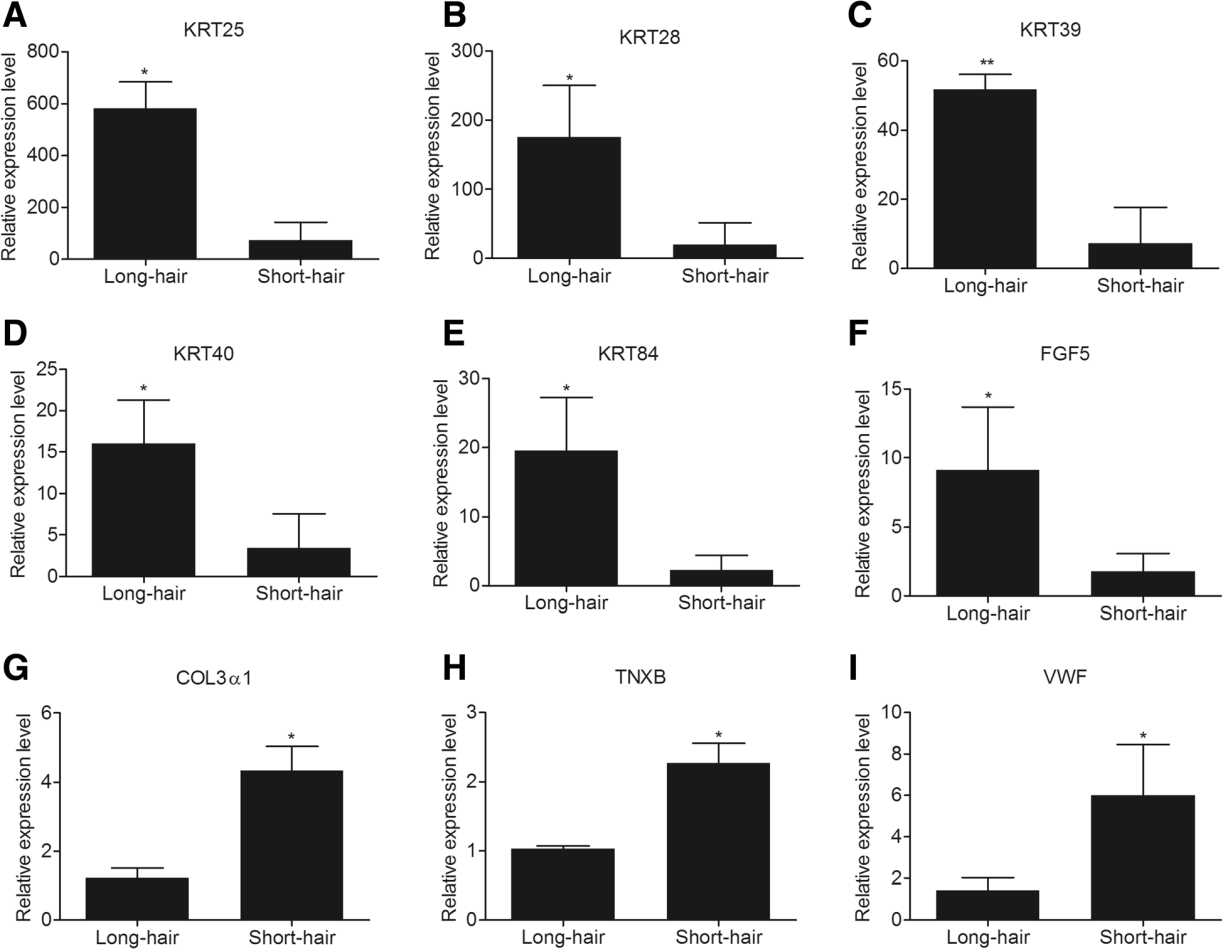
A q-PCR analysis of nine DEGs in the skin of short-hair and long-hair rabbits. **a** KRT25 **b** KRT28 **c** KRT39 **d** KRT40 **e** KRT84 **f** FGF5 **g** COL3α1 **h** TNXB **i** VWF. The skin tissues were from short-hair and long-hair rabbits at the sixth week after plucking. GAPDH was used as a reference gene to normalize q-PCR data. Bars represent the standard error. ** *P* < 0.01, * *P* < 0.05

To evaluate our DEG library, the relative expression levels of five DEGs from the KRT family (*KRT25*, *KRT28*, *KRT39*, *KRT40*, and *KRT84*) as well as *FGF5* and ECM-related genes (*COL3α1*, *TNXB*, and *VWF*) were analysed by q-PCR. As shown in Fig. 3 and Additional file 4: Figure S4, the results of RNA-Seq and q-PCR were similar. In general, the results of q-PCR validated the RNA-Seq results and demonstrated the reliability of our data. Furthermore, we also found that the expression profiles of the selected genes at the eighth week after plucking (Additional file 5: Figure S5) were similar to those at the sixth week.

### GO and KEGG analyses for DEGs between short-hair and long-hair rabbits

**Fig. 4:**
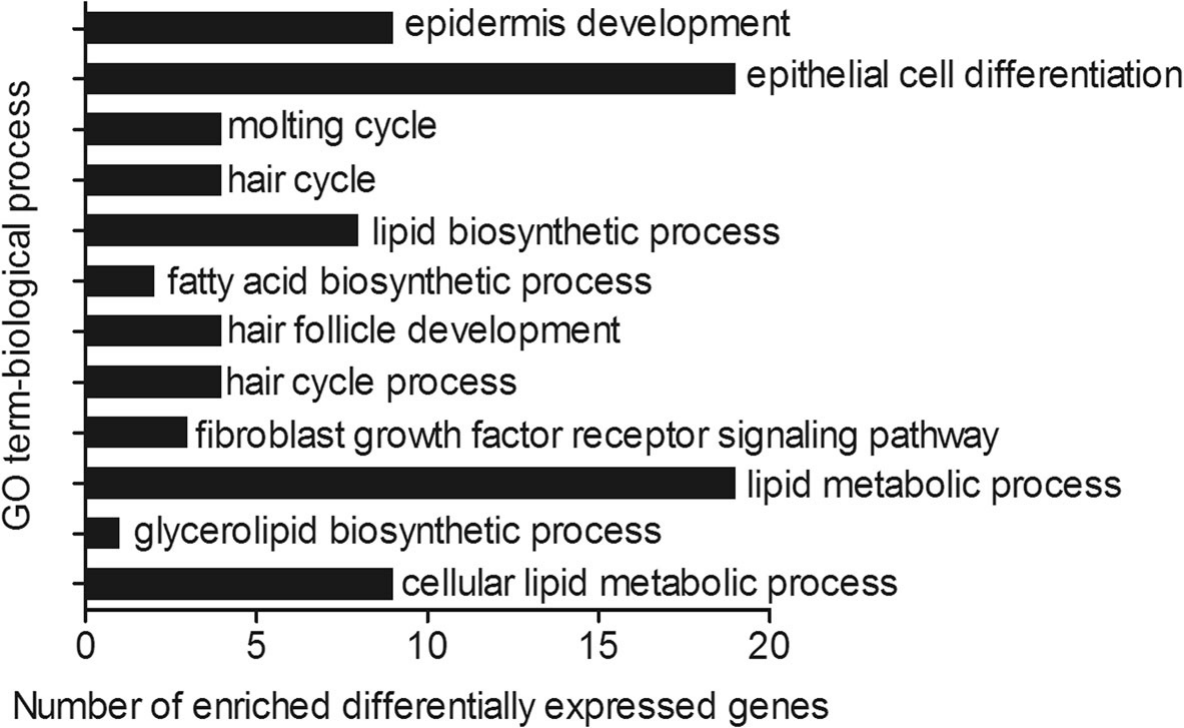
The significantly enriched GO terms of DEGs between short-hair and long-hair rabbits (P < 0.05). The hierarchical category of the GO terms is biological process. The x-axis represents the number of DEGs enriched in biological processes; y-axis represents the GO terms enriched by DEGs

Gene ontology (GO) and Kyoto Encyclopedia of Genes and Genomes (KEGG) were used for the functional analysis and signalling pathway annotations of these DEGs. The GO terms with the Q value ≤0.05 were considered to be significantly enriched. Several GO terms related to hair follicle development and lipid metabolism in the biological process category were found (Fig. 4, Additional file 6: Table S1). A comparison between short-hair and long-hair rabbits showed some DEGs involved in the biological processes including epidermis development (9 genes, Q = 0.00427), molting cycle (4 genes, Q = 0.01352), hair cycle (4 genes, Q = 0.01352), lipid biosynthetic process (8 genes, Q = 0.01829), hair follicle development (4 genes, Q = 0.02563), and so on (Additional file 6: Table S1). The GO analysis demonstrated large differences in hair follicle development and lipid metabolism between short-hair and long-hair rabbits. In addition, six important pathways related to the regulation of hair follicle development were identified (Table 4). Among them, the extracellular matrix (ECM)-receptor interaction and basal cell carcinoma pathway were significantly enriched between short-hair and long-hair rabbits (*P* < 0.05). Therefore, we focused on these two pathways for further characterisation. The DEGs enriched in the both pathways are listed in Table 4. In the ECM-receptor interaction, the expression of 15 DEGs, including the collagen genes (such as *COL1A2*, *COL3A1*, *COL5A2*, and *COL5A3*), laminin genes (such as *LAMA4* and *LAMC3*), integrin genes (such as *ITGB3* and *LOC103346304*), and tenascin genes (such as *TNN* and *TNXB*) was down-regulated in long-hair rabbits (Table 4). In the basal cell carcinoma pathway, 11 up-regulated genes, including *GLI1*, *PTCH1*, and *SHH*, were identified (Table 4).

**Table 4:** The DEGs enriched in the pathways related to skin development between short-hair and long-hair rabbits

Pathway	DEGs	
	Up	Down
ECM-receptor interaction		CHAD, COL1α2, COL3α1, COL5α2, COL5α3, FN1, ITGB3, LAMA4, LAMC3, LOC103346304, SPP1, THBS2, TNN, TNXB, VWF
Basal cell carcinoma	FZD5, GLI1, HHIP, LEF1, LOC100355594, PTCH1, PTCH2, SHH, TCF7, WNT11, WNT5A	
Hedgehog signalling pathway	GLI1, HHIP, LOC100341681, PTCH1, PTCH2, SHH, WNT11, WNT5A	GAS1,
TGF-beta signalling pathway	BAMBI, BMP7, LOC100008826, PPP2R1B, LOC100341360, LOC103347033, SMAD6, SMAD7,	SMAD9, TGFBR2, TGFB3, INHBA,
Wnt signalling pathway	BAMBI, FZD5, LEF1, LOC100341681, LOC100355594, TCF7, WNT11, WNT5A	DAAM2, DKK2,
Notch signalling pathway		DTX3L, DTX4

### Alternative splicing and single nucleotide polymorphisms analyses

In the present study, we detected various splicing events, including alternative 5′ first exon (TSS), alternative 3′ last exon (TTS), alternative exon ends (AE), intron retention (IR), and skipped exon (SKIP) (Additional file 7: Figure S6). The number of alternative splicing events and types was similar between the long-hair and short-hair rabbits. The TSS and TTS, which occur when two or more splice sites at 5′ first or 3′ last exon are present, are the most frequently observed events, accounting for approximately 41 and 31% of alternative splicing events in rabbits, respectively. SKIP is the next most common event in AS accounting for approximately 17% of alternative splicing events. IR, whereby an intron is retained in the resulting mature mRNA, is barely detected, accounting for approximately 1.6% of the AS events. These results showed that the number of each AS event was similar between the short-hair and long-hair rabbits.

In addition, we compared the sequence data with known reference sequences using the TopHat2 software, and then identified potential SNPs. A total of 726,852 and 852,149 SNPs were detected in long-hair and short-hair rabbits, respectively (Additional file 8: Table S2). Compared with that in the long-hair rabbits, more SNPs were identified in short-hair rabbits, demonstrating the differences in SNPs during skin development in short-hair and long-hair rabbits. Approximately 75% SNP sites belonged to the transition type and 25% SNP sites were transversion type. The proportion of heterozygosity SNPs was different in each sample, accounting for 30.90–34.98%. Meanwhile, more SNP sites were found in genic regions in each sample. As presented in Additional file 9: Figure S7, the proportion of SNPs in the intron region was the highest.

## Discussion

Large phenotypic differences exist with respect to hair length between short-hair and long-hair rabbits. The cycle of hair follicles comprises three distinct phases—growth, regression, and resting phases [2, 8, 9]. These stages can be distinguished according to hair follicle morphology. In our previous studies, we reconstructed the follicle cycle of rabbit hair growth by plucking hairs and analysed the hair growth rhythm of short-hair and long-hair rabbits [29, 30]. The results showed a linear upward trend from the fourth to eleventh week in different types of hair from long-hair rabbits, and the hair of short-hair rabbits exhibited rapid growth before the sixth week and remained stable after 6 weeks and suggested different hair growth rates and hair cycles between the two populations. In the present study, we determined the follicle morphology of short-hair and long-hair rabbits at week four, six, and eight after plucking. The results demonstrated that the hair follicles were still in the anagen phase until the eighth week. The regression phase of hair follicle development in long-hair rabbits needs further studies; the hair follicles in short-hair rabbits began to degenerate from the fourth to sixth week and entered the resting phase from the sixth to eighth week. Hair growth is fuelled by bulge stem cells, which are activated at the start of the anagen phase by the dermal papilla [31]. In the present study, long growth phase of hair follicles in long-hair rabbits continuously promoted hair growth. During the catagen phase, hair follicle stem cells in the outer root sheath and the germinal matrix undergo apoptosis, and the lower two-third of hair follicles regress [9]. In the telogen phase, hair follicle stem cells are relatively quiescent, inhibiting hair growth [32]. These findings were consistent with our results, which showed that hair follicles at the sixth week were in the catagen phase and entered the telogen phase when they were at the eighth week after plucking in short-hair rabbits, resulting in the cessation of hair growth.

The present study also provides an opportunity to compare the transcriptome of skin tissue between short-hair and long-hair rabbits by high-throughput sequencing to analyse the molecular mechanism associated with differences in hair growth. The transcriptome data obtained are useful to elucidate the different mechanisms of hair growth in the skin of long-hair and short-hair rabbits. It has been reported that > 18 M reads from RNA-Seq are required for each sample to attain a saturated state for expression analysis [32, 33]. More than 36 M clean reads were obtained from each sample in the present study, and the quality control assay of our data revealed that the RNA-Seq data were well qualified (Table 1, Additional file 1: Figure S1). The analyses of heatmaps and PCA of DEGs revealed a larger number of significantly DEGs in the skin tissue of short-hair and long-hair rabbits at the sixth week after plucking hairs. These genes might play crucial roles in regulating hair follicle development and hair growth, and their differential expression might be the reason for difference in hair length between short-hair and long-hair rabbits. Ten genes encoding the structural proteins of wool were detected (Table 3). Keratin intermediate filament (KRT-IF) and keratin-associated protein genes encode the majority of wool and hair proteins [34, 35]. The wool follicle-related genes *KRT34*, *KRT38*, and *KRT39* were expressed only in the cortex, and *KRT40* and *KRT84* were expressed in the fibre cuticle [34]. Ovine *KRT25-KRT28*, which encode type I KRTs in the inner root sheath, have also been described [34]. The primary function of KRTs is to protect epithelial cells from apoptosis and mechanical or nonmechanical stresses that can lead to cell death [36, 37]. However, in the present study, a few genes encoding keratin-associated protein were differentially expressed in the two populations. Interestingly, the expression of 10 genes of the KRT family was up-regulated in the skin tissue of long-hair rabbit, suggesting that the KRT genes might contribute to the continuous activity of hair follicle in the long-hair rabbit. We postulated that the 10 KRT genes might also be useful in promoting hair growth. In earlier reports, FGF5 has been identified as an inhibitor of hair growth in various mammals. FGF5 induced regression of the human hair follicle [38] and knock-out and disruption of *FGF5* using the CRISPR/Cas9 system in goat resulted in longer fibres [39, 40]. However, there is a recent report on rabbits indicating that *FGF5* was also up-regulated in the long wool group (Wan line Angora rabbit) and down-regulated in the short wool group (Chinchilla Rex rabbit and White Rex rabbit) [41], which was consistent with our findings of RNA-seq and q-PCR, showing that the *FGF5* gene was up-regulated in the skin of long-hair rabbit. Previous studies also showed that the polypeptide of FGF5s translated from an alternatively spliced variant of *FGF5* mRNA suppressed the activity of FGF5, increasing hair growth [42, 43]. We hypothesized that FGF5s of *FGF5* mRNA suppressed the activity of FGF5 and promoted hair growth in the rabbit. Further studies are required to investigate the roles of alternatively spliced variants of the *FGF5* genes in hair length. In addition, the nine genes were also selected for validation by q-PCR to evaluate our DEG library. All the selected genes showed similar expression patterns between RNA-Seq and q-PCR, demonstrating the reliability of our data. An additional expression analyses of the selected genes showed that their expression profiles at the eighth week after plucking were similar to those at the sixth week. The hair follicles of short-hair and long-hair rabbits at the eighth week were in the telogen and the anagen phase, respectively. The finding further demonstrated that the genes played important roles in regulating hair follicle development and hair growth.

The GO analysis showed that a large proportion of DEGs were significantly enriched in the biological processes such as epidermis development, epithelial cell differentiation, hair follicle development, and lipid metabolism process. The results demonstrated that the DEGs are potential regulators of hair follicle development and lipid metabolism in rabbits.

In addition, a number of DEGs were also found to be significantly enriched in extracellular matrix (ECM)-receptor interaction and basal cell carcinoma pathway. The extracellular matrix in the ECM-receptor interaction is a complex mixture of structural and functional macromolecules, including fibrous proteins (collagen, fibronectin, and laminin) [44, 45]. Different collagens form different ECM components, for example, collagens 1, 3, and 5 form the fibrils, collagen 6 forms the microfibril, and collagen 4 forms the basal membrane [46]. The amount of ECM per cell contributes to the volume of the dermal papilla [47]. The previous study showed that many of the ECM-related genes, including *collagen*, *laminin*, and *fibronectin* were differentially expressed between primary and secondary hair follicle dermal papilla cells, resulting in an enlarged dermal papilla [48]. Dermal papillas control the number of matrix cells and, thus, the character and size of the hair follicle and its shaft [49]. In the present study, the differential expression of *COL1α2*, *COL3α1*, *COL5α2*, and *COL5α3* suggested a relation to the size difference of the hair follicle and its shaft between the two populations and the PCA further showed that *COL1α2* and *COL3α1* were important regulators of hair follicle development. The differential expression of other ECM-related genes, such as *ITGB3*, *LAMA4*, *LAMC3*, *VWF*, and *TNXB*, also shows the difference in the ECM structure between short-hair and long-hair rabbits. The results of the present study also showed that the expression of *GLI1*, *SHH*, and *PTCH1* involved in the basal cell carcinoma pathway was up-regulated in short-hair rabbits. It has been reported that the inhibition of GLI1 induces cell-cycle arrest and enhances apoptosis in brain glioma cell lines [50]. SHH and PTCH1 are also key components of the Hedgehog pathway, which primarily regulates the genes involved in cell growth, proliferation, survival, and apoptosis [51]. Hair follicle genes were also found to be involved in cell differentiation, proliferation, apoptosis, and growth in Hu sheep lambskin [52]. Therefore, the differential expression of *GLI1*, *SHH*, and *PTCH1* suggests the difference in hair follicle cycle and apoptosis in the two populations, and their roles in the regulation of hair follicle cycle and apoptosis in rabbits. WNT signals are required for the initiation of hair follicle development [14]. It has also been reported that LEF-1 is involved in controlling the development of whisker follicles [53], and WNT5a inhibits the growth of hair follicles in mice [54]. It has been reported that Wnt, Shh, TGF-b and Notch signalling pathways may be involved in hair follicle development and cycling in cashmere goats [55]. The results of the present study showed that LEF-1, WNT5a, and TCF7 involved in the WNT signalling pathway were significantly up-regulated in long-hair rabbits. Further, the Notch signalling pathway regulates hair cell differentiation in the cochlea of mammals [56] and mediates hair cell regeneration partially depending on Wnt signalling [57]. In the present study, we found the upregulation of BAMBI and SMAD7 in the TGF-β signalling pathway in long-hair rabbits; they are the key regulators of hair cycle [7]. Overall, the candidate genes identified in the present study might therefore serve as potential regulators of hair length in rabbits.

Alternative splicing and SNPs can increase protein complexity and cause diversity in biological traits. Alternative splicing is one of the major mechanisms contributing to protein diversity [58]. Although AS is widespread in eukaryotes, we tend to underestimate its proportion. The changes in AS might therefore represent a major source of species-specific differences [59–61]. In this study, we detected five primary alternatively spliced events and analysed the number of each AS event (Additional file 7: Figure S6). The results showed that the number of TSS and TTS events was the highest and the IR event was the least in rabbits. Most genomic variations are attributable to the SNPs, and therefore, offer the highest resolution to track disease-related genes and population history [62, 63]. A total of 231,653–317,941 SNPs were found to specifically match the genome of *Oryctolagus cuniculus* in six rabbit samples. Compared with the intergenic SNPs, more SNPs were found in genic regions where most SNPs are located in the intron regions. The SNPs in or near genes explain more variations than the SNPs between genes for complex traits [64, 65]. These results imply that the diversity in regulation of complex traits, which can be by either genic regions or nearby gene regions, and even by intergenic region. The specific roles of AS and SNPs in regulating hair length of short-hair and long-hair rabbits need further study.

## Conclusions

In conclusion, we identified differences in the histology and transcriptome profile of skin between short-hair and long-hair rabbit. The histological analysis showed different hair cycles and growth rate between the two populations. The differences in the transcriptome profile of the two populations were consistent with the differences observed in morphological traits. RNA-Seq enabled us to obtain candidate genes involved in hair follicle development, lipid metabolism, and apoptosis. The expression of differentially expressed lipid metabolic and apoptotic genes in the skin of rabbits can be used as a novel trait with the potential to alter hair length. Further studies are required to investigate the roles of candidate genes in hair length to improve rabbit breeding programmes.

## Methods

### Animals

**Fig. 5:**
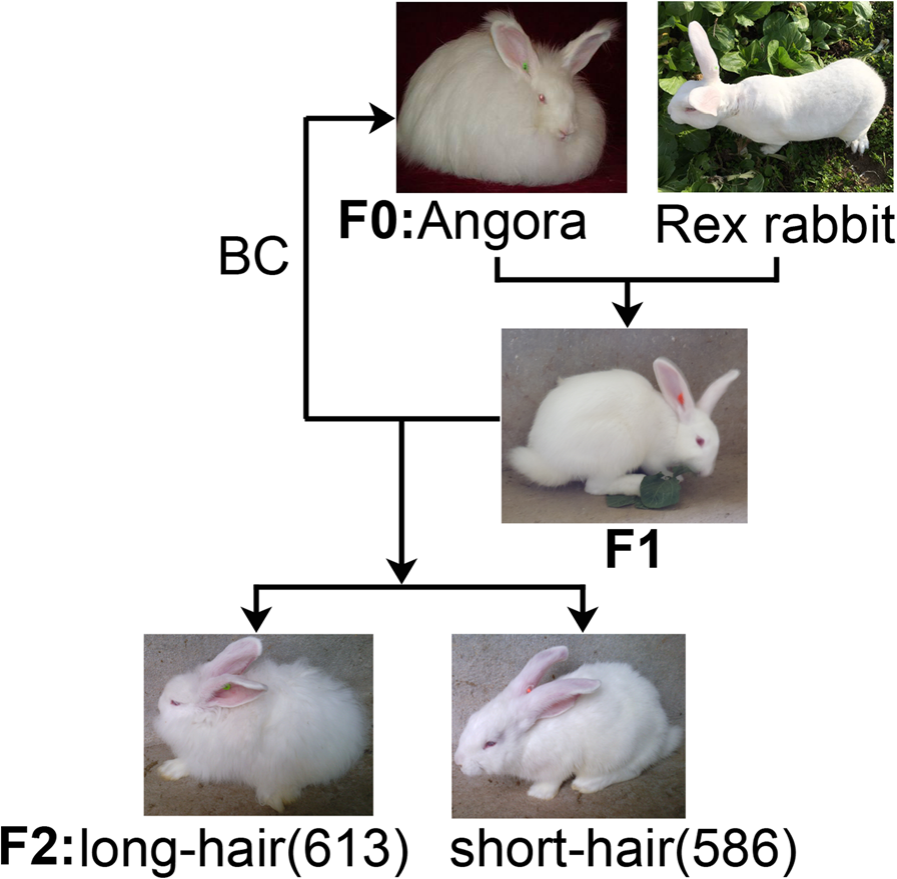
Hair length trait segregation in the family. Two phenotypic traits segregate in the F2 population: long-hair, with recessive-type allele and short-hair, with wild-type and mutated alleles. All the images of rabbits are our own and photographed by our researchers

All rabbits were procured from the rabbit farm in the Animal Husbandry Institute of Anhui Academy of Agriculture Sciences, Hefei, Anhui, China. The rabbits were raised and managed under the same condition, feeding to appetite; drinking water was supplied via an automatic drinking bowl. A cross between the Wan Strain Angora rabbit and Rex rabbit was carried out to produce the F1 generation (Fig. 5). The F1 generation was then backcrossed (BC) with the Wan Strain Angora rabbit, which produced F2 rabbits. Two phenotypic traits segregated in the families; two hair length characters (586 short-hair and 613 long-hair rabbits). The segregation ratio of hair length in F2 generation was consistent with Mendel heredity principles (1:1, χ^2^ = 0.564, *P* = 0.436). Normal rabbit fur is composed of three different types of hairs including coarse hairs, awn hairs, and fine hairs [66, 67]; the length of the different hair types was measured at the tenth week after plucking (Additional file 10: Figure S8). Finally, three female short-hair and long-hair rabbits were selected for the present study, respectively.

### Sample collection, preparation, and histological examination

Rabbits were given anesthesia through an ear vein injection of 0.7% pentobarbital sodium (6 ml/kg) before sampling. Skin tissue samples (1 cm^2^) from the back of each rabbit were collected at week four, six and eight after plucking hairs for histological analysis. The skin samples were fixed with 4% formaldehyde (40% formaldehyde solution and distilled water mixed at a 1:9 ratio) solution. Cross sections of the fixed samples were washed with running water, dehydrated using an ethyl alcohol series, cleaned in xylene, and embedded in paraffin wax. The specimens were sectioned to a thickness of 4 μm using a Leica RM2235 microtome (Leica, Wetzlar, Germany). Transverse and vertical cross-sections of the fixed and paraffin-embedded samples were stained with HE, examined and photographed using an Olympus BX51 biomicroscope (Olympus Optical Company, Tokyo, Japan).

### cDNA library construction and sequencing

Under anesthesia, skin samples from the back were collected at the sixth week after plucking the hair, when the wool cycle of long-hair rabbit is expected to be in the anagen phase and that of short-hair rabbit is expected to be in catagen phase, frozen in liquid nitrogen immediately, and stored at − 80 °C prior to RNA extraction. The total RNA was extracted from the skin samples of long-hair rabbits (designated as L1, L2, and L3) and short-hair rabbits (designated as S1, S2, and S3) using TRIzol reagent (Invitrogen, Carlsbad, CA, USA) according to the manufacturer’s instructions. The samples were sent to the Beijing Biomarker Technologies Company. The quality of RNA was evaluated using the RNA integrity number value using the Agilent 2100 Bioanalyzer (Agilent, Santa Clara, CA, USA). The mRNA was purified, fragmented, and converted to cDNA, adapters were ligated to the end of double-stranded cDNA, and libraries were created by polymerase chain reaction (PCR) using the Illumina TruSeq RNA Sample Preparation Kit (Illumina, San Diego, CA, USA) according to manufacturer’s protocols. The libraries constructed were quantified with Qubit2.0 and the insert size library was determined using Agilent 2100. Six independent paired-end libraries were sequenced on an Illumina HiSeq 2500 (Illumina, San Diego, CA, USA) system according to the manufacturer’s instructions. Above 10.50 Gb clean reads per sample were generated for the genome-wide transcriptomic analyses.

### Mapping and assembling

The trimmed reads were assembled and mapped to the reference genome (https://www.ncbi.nlm.nih.gov/genome/?term=Rabbit) by performing alignments using the TopHat2 [68] software. The AS and SNPs were analysed using the Cufflinks [69] and GATK [70] software, respectively.

### Differential gene expression analysis

The values of fragments per kilobase of transcript per million fragments mapped (FPKM) [71] were generated and used to identify the expression level of genes in each rabbit sample. Differential expression analysis was performed with DESeq, an R package [72]. The *P*-value corresponds to a differential gene expression test in which the false discovery rate (FDR) was used to determine the threshold of P-value in multiple tests. We filtered the differentially expressed genes (DEGs) with the standards (Fold Change ≥2 and FDR < 0.01). The gene ontology (GO) analysis was employed to analyse the functions of the DEGs. All the DEGs were mapped to the GO terms in the database (http://www.geneontology.org/). The Kyoto Encyclopedia of Genes and Genomes (KEGG) database was used to perform pathway enrichment analysis of the DEGs. Cluster 3.0 [73] was used for the clustering analysis.

### Quantitative real-time PCR

To verify the RNA-Seq results, a set of six candidate genes were selected from the list of DEGs and the relative expression level of each gene was evaluated by quantitative real-time PCR (q-PCR). The PCRs were performed for each individual using 1.0 μg of residual RNA from the original extraction described above. The first-strand cDNA was synthesised using EasyScript One-Step gDNA Removal and cDNA Synthesis SuperMix (Transgen, Beijing, China). The q-PCR was carried out in a LightCycler 96 (Roche, Basel, Switzerland) using TransStart Green qPCR SuperMix (Transgen, Beijing, China). The following conditions were used for amplification: 94 °C for 30 s, followed by 40 cycles of 94 °C for 5 s, 62 °C for 35 s, and a melt curve stage of 97 °C for 10 s, 65 °C for 1 min, and 97 °C for 1 s. The primers for the q-PCR are listed in Additional file 11: Table S3. The *GAPDH* gene was used as a constitutive expression control in these experiments. The relative expression level of each gene was estimated by the 2^-ΔΔCT^ method [74]. The q-PCR analysis was performed in triplicates for each sample.

### Statistical analyses

Excel 2010 (Microsoft) was used to analyse the data. The data are presented as mean ± standard deviation (SD). Student’s *t*-test was used for statistical comparisons. The results with a *P* value < 0.05 were considered to be statistically significant.

## Additional files

Additional file 1: **Figure S1.** Quality control of RNA-seq data. **A** L1 **B** L2 **C** L3 **D** S1 **E** S2 **F** S3. (PDF 236 kb)

Additional file 2: **Figure S2.** The numbers of annotated genes with different expression levels against the range of FPKM values. (PDF 36 kb)

Additional file 3: **Figure S3.** List of up-regulated and down-regulated genes in the comparison of short-hair and long-hair rabbits. (PDF 118 kb)

Additional file 4: **Figure S4.** The results of RNA-seq between long-hair and short-hair rabbits. **A** KRT25 **B** KRT28 **C** KRT39 **D** KRT40 **E** KRT84 **F** FGF5 **G** COL3α1 **H** TNXB **I** VWF. (PDF 154 kb)

Additional file 5: **Figure S5.** A q-PCR analysis of the relative expression levels of nine DEGs in the skin of short-hair and long-hair rabbits at the eighth week after plucking by q-PCR. **A** KRT25 **B** KRT28 **C** KRT39 **D** KRT40 **E** KRT84 **F** FGF5 **G** COL3α1 **H** TNXB **I** VWF. (PDF 175 kb)

Additional file 6: **Table S1.** Significantly enriched GO terms in the biological process category of DEGs between short-hair and long-hair rabbits (*P* < 0.05). (PDF 13 kb)

Additional file 7: **Figure S6.** The number of alternative splicing events of six samples. **A** L1 **B** L2 **C** L3 **D** S1 **E** S2 **F** S3. (PDF 144 kb)

Additional file 8: **Table S2.** Analyses of SNP sites of six samples. (PDF 12 kb)

Additional file 9: **Figure S7.** Analyses of SNPs annotation. **A** L1 **B** L2 **C** L3 **D** S1 **E** S2 **F** S3. (PDF 355 kb)

Additional file 10: **Figure S8.** Hair length of the short-hair and long-hair rabbits at the tenth week after plucking. Fibres are divided into three categories, including coarse, fine, and awn fibres. (PDF 107 kb)

Additional file 11: **Table S3.** Primers for qPCR. F^1^, forward primer. R^2^, reverse primer. (PDF 95 kb)

## Abbreviations

AS: Alternative splicing
DEGs: Differentially expressed genes
ECM: Extracellular matrix
FDR: False discovery rate
FPKM: Fragments per kilobase of transcript per million fragments mapped
GO: Gene ontology
HE: Haematoxylin-eosin
KEGG: Kyoto Encyclopedia of Genes and Genomes
KRT: Keratin
PCA: Principal component analysis
q-PCR: Quantitative real-time PCR
RNA-Seq: RNA sequencing
SD: Standard deviation
SNP: Single nucleotide polymorphisms
