# Supplementary figures and images for: Analyses of histological and transcriptome differences in the skin of short-hair and long-hair rabbits
Source: BMC Genomics. 2019 Feb 15;20:140. doi: 10.1186/s12864-019-5503-x (PMC6377753; doi:10.1186/s12864-019-5503-x)

Figure S1

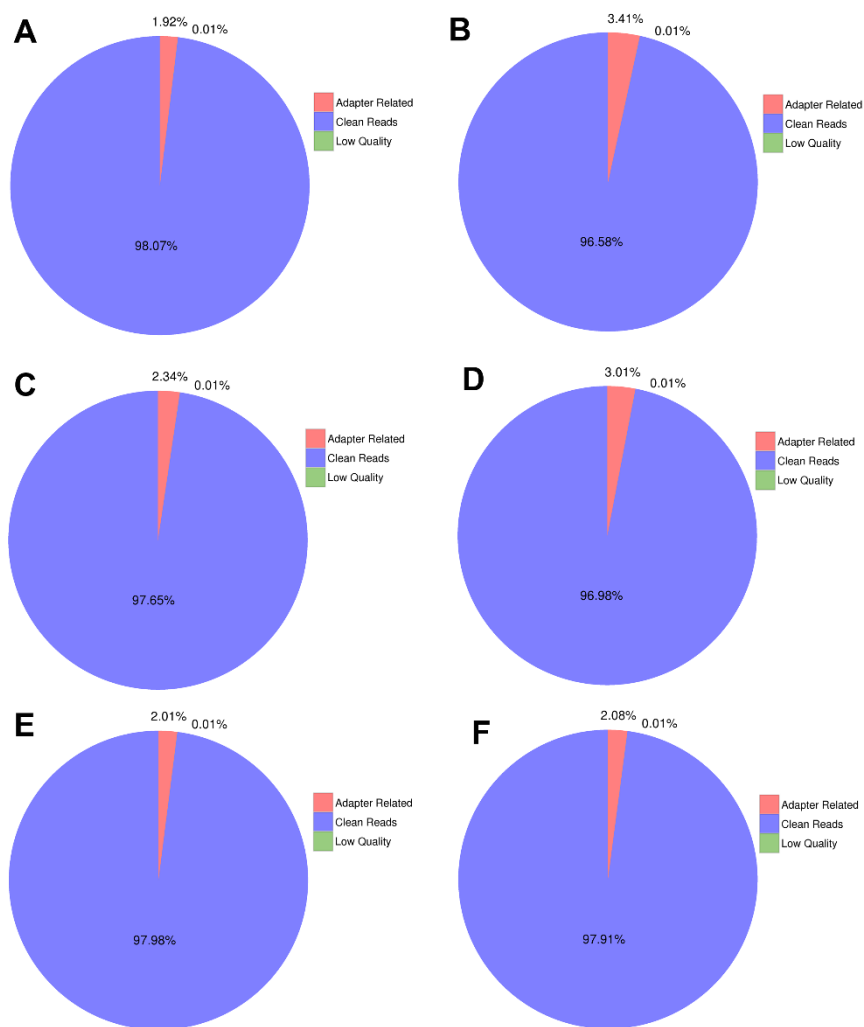

Supplement: Supplementary file 1 — Figure S1. Quality control of RNA-seq data. A L1 B L2 C L3 D S1 E S2 F S3. (PDF 236 kb) [file 12864_2019_5503_MOESM1_ESM.pdf]

Figure S2

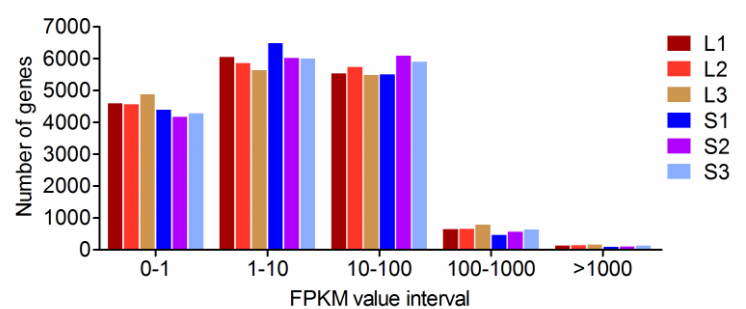

Supplement: Supplementary file 2 — Figure S2. The numbers of annotated genes with different expression levels against the range of FPKM values. (PDF 36 kb) [file 12864_2019_5503_MOESM2_ESM.pdf]

Figure S3

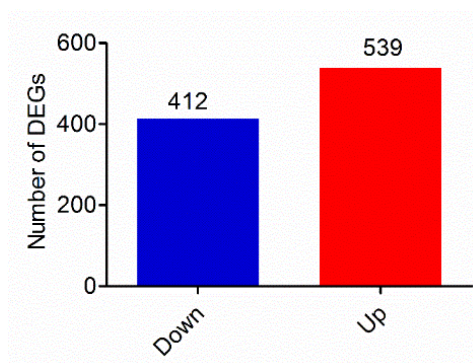

Supplement: Supplementary file 3 — Figure S3. List of up-regulated and down-regulated genes in the comparison of short-hair and long-hair rabbits. (PDF 118 kb) [file 12864_2019_5503_MOESM3_ESM.pdf]

Figure S4

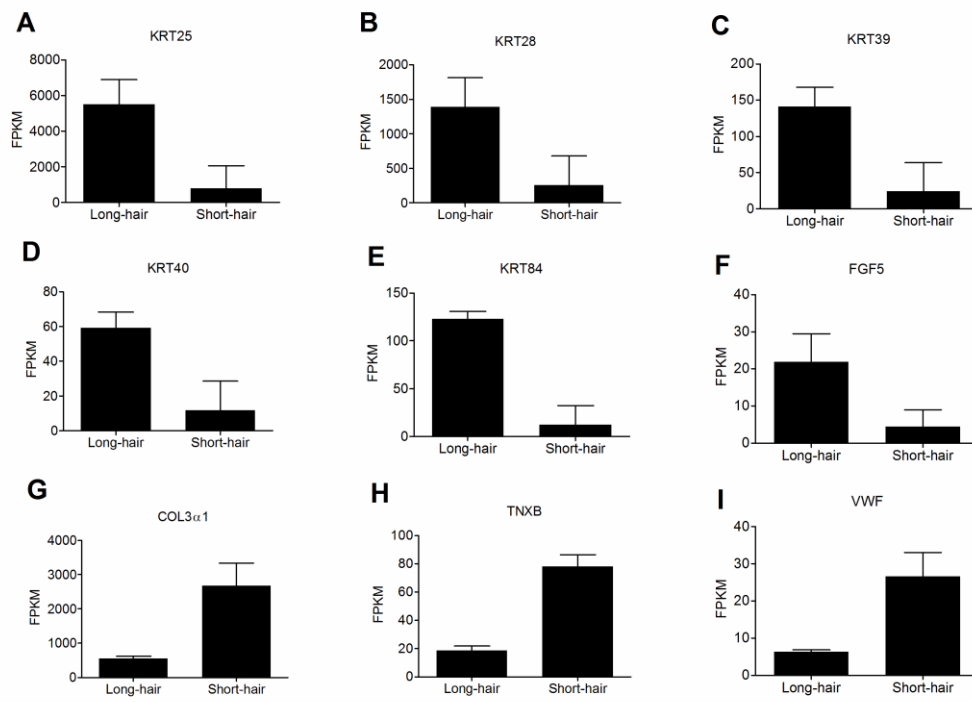

Supplement: Supplementary file 4 — Figure S4. The results of RNA-seq between long-hair and short-hair rabbits. A KRT25 B KRT28 C KRT39 D KRT40 E KRT84 F FGF5 G COL3α1 H TNXB I VWF. (PDF 154 kb) [file 12864_2019_5503_MOESM4_ESM.pdf]

Figure S5

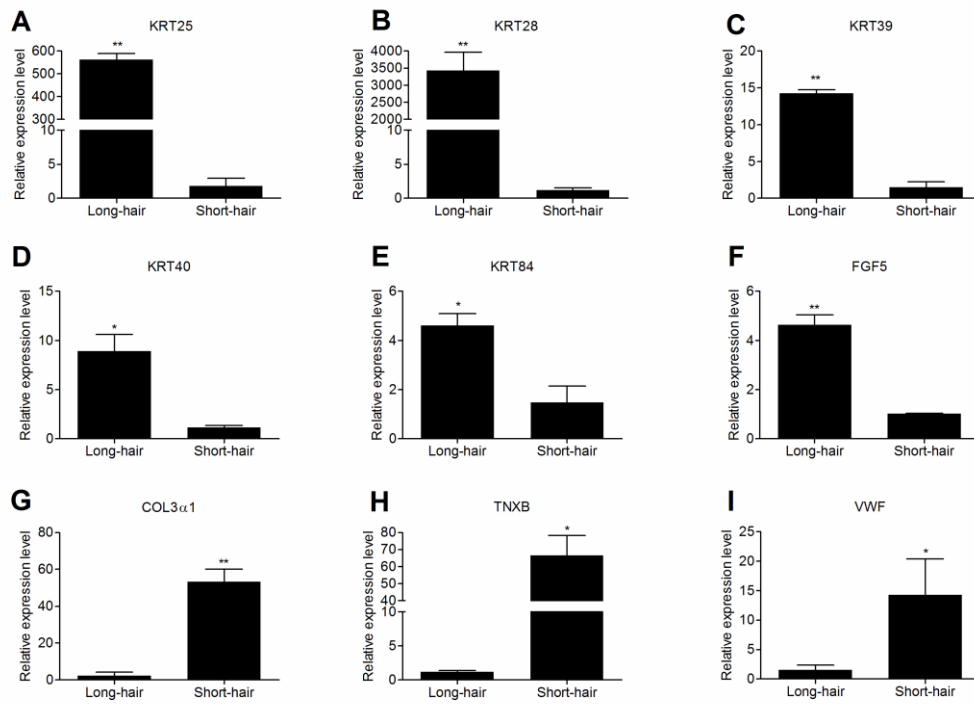

Supplement: Supplementary file 5 — Figure S5. A q-PCR analysis of the relative expression levels of nine DEGs in the skin of short-hair and long-hair rabbits at the eighth week after plucking by q-PCR. A KRT25 B KRT28 C KRT39 D KRT40 E KRT84 F FGF5 G COL3α1 H TNXB I VWF. (PDF 175 kb) [file 12864_2019_5503_MOESM5_ESM.pdf]

Figure S6

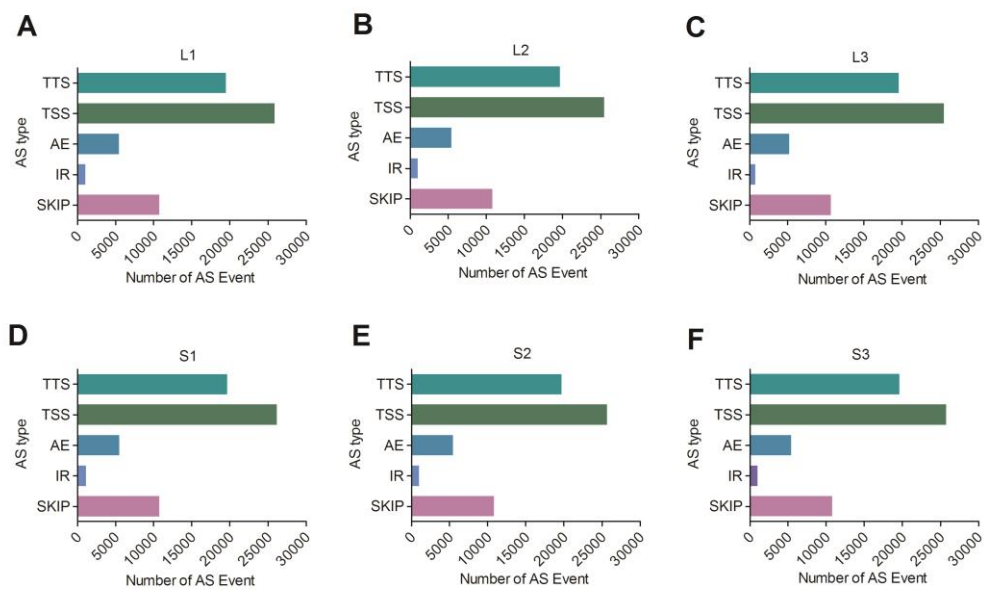

Supplement: Supplementary file 7 — Figure S6. The number of alternative splicing events of six samples. A L1 B L2 C L3 D S1 E S2 F S3. (PDF 144 kb) [file 12864_2019_5503_MOESM7_ESM.pdf]

Figure S7

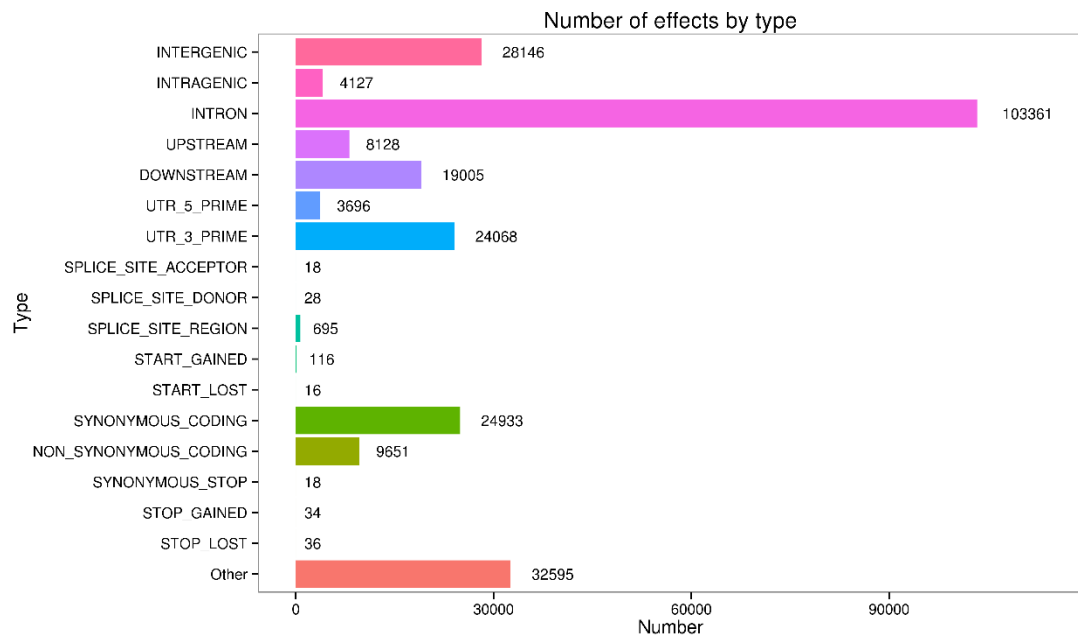

A

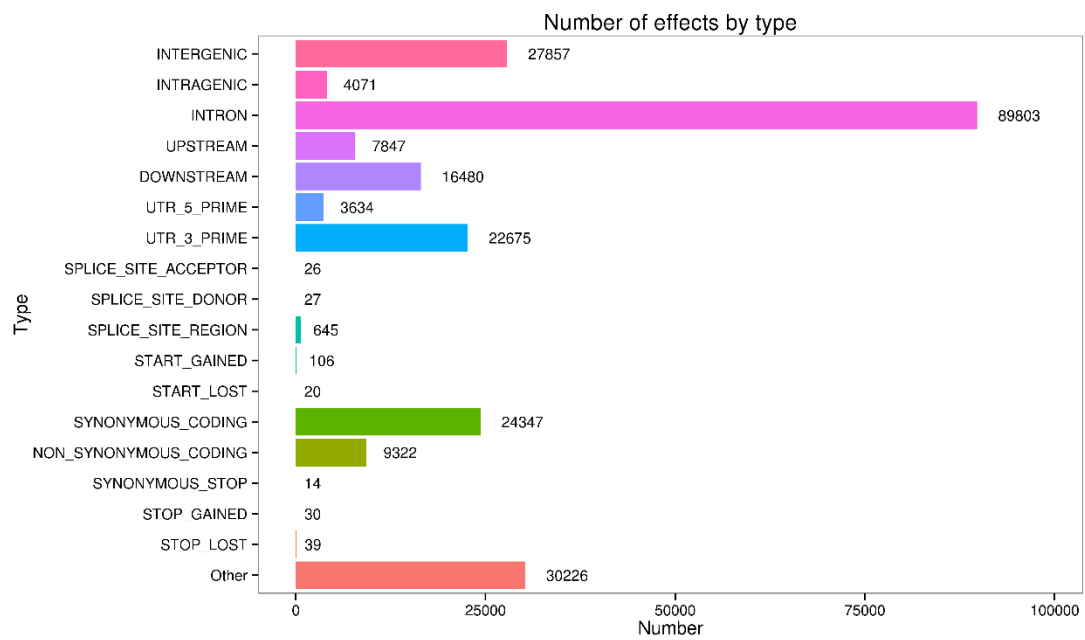

B

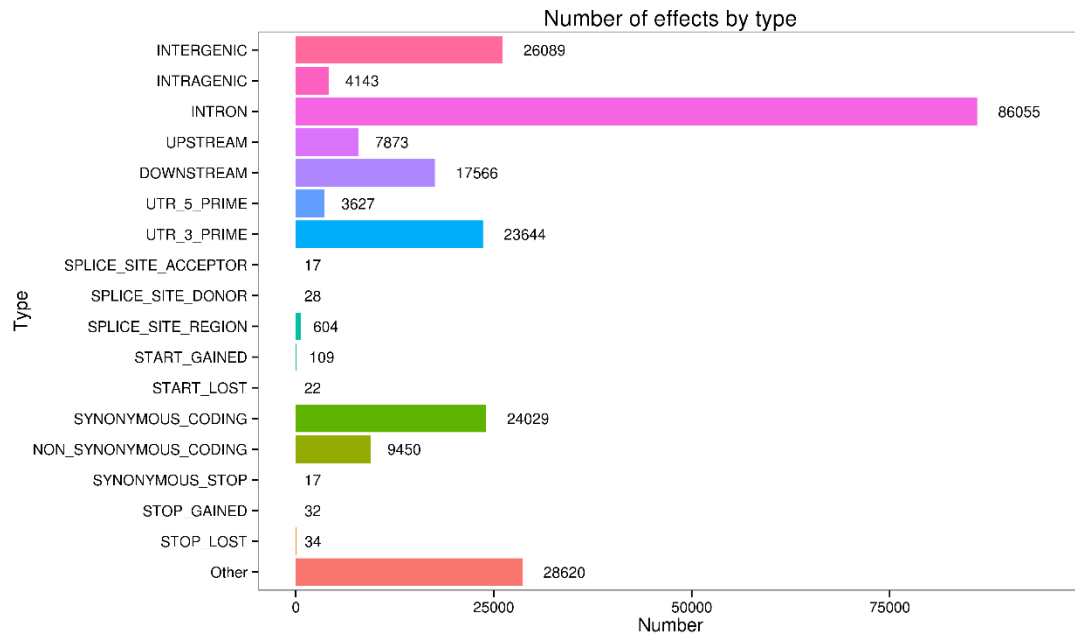

C

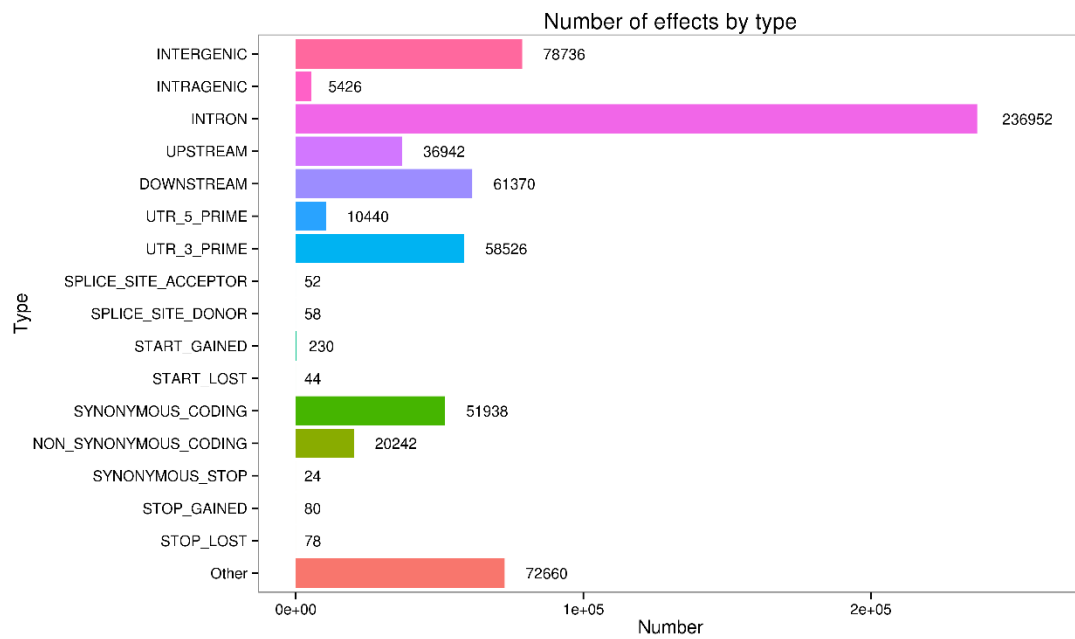

D

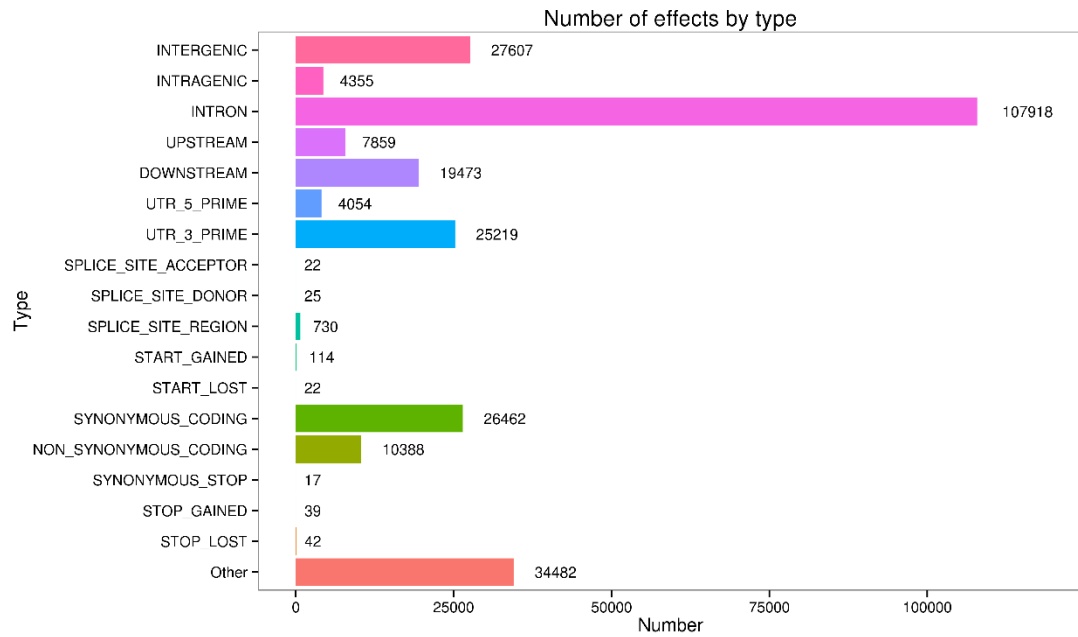

**E**

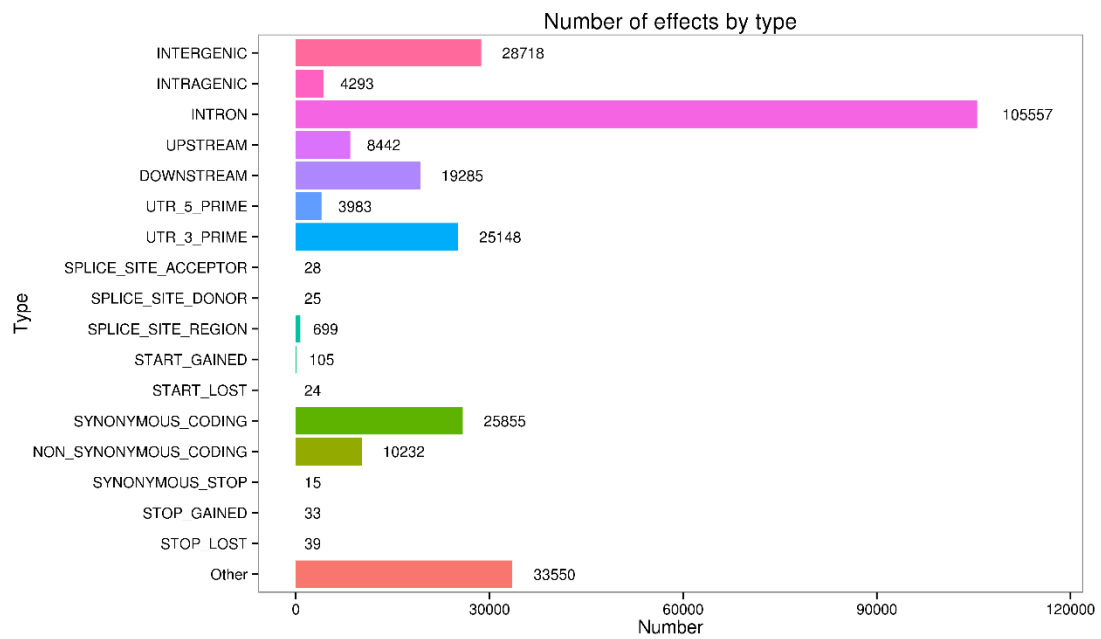

**F**

Supplement: Supplementary file 9 — Figure S7. Analyses of SNPs annotation. A L1 B L2 C L3 D S1 E S2 F S3. (PDF 355 kb) [file 12864_2019_5503_MOESM9_ESM.pdf]

Figure S8

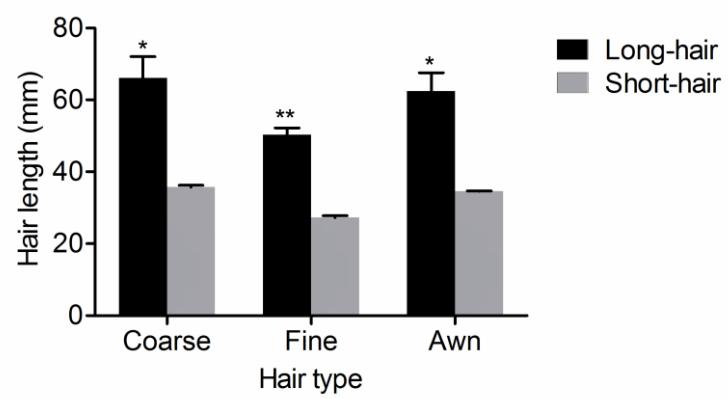

Supplement: Supplementary file 10 — Figure S8. Hair length of the short-hair and long-hair rabbits at the tenth week after plucking. Fibres are divided into three categories, including coarse, fine, and awn fibres. (PDF 107 kb) [file 12864_2019_5503_MOESM10_ESM.pdf]
