# Supplementary material for: Analyses of histological and transcriptome differences in the skin of short-hair and long-hair rabbits
Source: BMC Genomics. 2019 Feb 15;20:140. doi: 10.1186/s12864-019-5503-x (PMC6377753; doi:10.1186/s12864-019-5503-x)
Supplement: Supplementary file 6 — Table S1. Significantly enriched GO terms in the biological process category of DEGs between short-hair and long-hair rabbits (P < 0.05). (PDF 13 kb) [file 12864_2019_5503_MOESM6_ESM.pdf]

Table S1

GO.ID	Term	p Value	Number of DEGs
GO:0008544	epidermis development	0.00427	9
GO:0030855	epithelial cell differentiation	0.00428	16
GO:0042303	molting cycle	0.01352	4
GO:0042633	hair cycle	0.01352	4
GO:0008610	lipid biosynthetic process	0.01829	8
GO:0006633	fatty acid biosynthetic process	0.02484	2
GO:0001942	hair follicle development	0.02563	4
GO:0022405	hair cycle process	0.02563	4
GO:0008543	fibroblast growth factor receptor signalling pathway	0.03275	3
GO:0006629	lipid metabolic process	0.03689	19
GO:0045017	glycerolipid biosynthetic process	0.03899	1
GO:0044255	cellular lipid metabolic process	0.04482	9
