# Supplementary material for: Analyses of histological and transcriptome differences in the skin of short-hair and long-hair rabbits
Source: BMC Genomics. 2019 Feb 15;20:140. doi: 10.1186/s12864-019-5503-x (PMC6377753; doi:10.1186/s12864-019-5503-x)
Supplement: Supplementary file 8 — Table S2. Analyses of SNP sites of six samples. (PDF 12 kb) [file 12864_2019_5503_MOESM8_ESM.pdf]

Table S2

Samples	SNP Number	Genic SNP	Intergenic SNP	Transition	Transversion	Heterozygosity
L1	258,338	205,438	52,900	75.13%	24.87%	34.98%
L2	236,861	186,697	50,164	75.07%	24.93%	30.90%
L3	231,653	182,480	49,173	74.92%	25.08%	33.82%
S1	317,941	252,257	65,684	74.90%	25.10%	32.66%
S2	268,516	216,072	52,444	75.38%	24.62%	34.75%
S3	265,692	211,624	54,068	75.43%	24.57%	34.60%
