# Supplementary material for: Analyses of histological and transcriptome differences in the skin of short-hair and long-hair rabbits
Source: BMC Genomics. 2019 Feb 15;20:140. doi: 10.1186/s12864-019-5503-x (PMC6377753; doi:10.1186/s12864-019-5503-x)
Supplement: Supplementary file 11 — Table S3. Primers for qPCR. F1, forward primer. R2, reverse primer. (PDF 95 kb) [file 12864_2019_5503_MOESM11_ESM.pdf]

Table S3

Primer	Sequences of primers (5´-3´)	TM (℃)	Size (bp)
KRT25-F[1]	GCAATTAACCTGAGACGCGAC	62	152
KRT25-R[2]	GGGGAGATCTGCGATTGATT		
KRT28-F	CAAAGGGCTTCGGATTAGGA	62	124
KRT28-R	GCGTGAATCCGTGATGAAAG		
KRT39-F	CGCGACAGGTGTGGAATTTA	62	138
KRT39-R	TTGGCATGAGGTTGAAGAGG		
KRT40-F	TCTCCTTTCAGCTCATTTTGGTA	62	152
KRT40-R	AACAACTGCTTTGCCATCAGG		
KRT84-F	GGGACCAGTTCAGCACCTTA	62	173
KRT84-R	CTCCGGGACCTGTGGATC		
FGF5-F	GGCTTGGAGCAGAGCAGTT	62	99
FGF5-R	CGGGTAGATCTGCAGATGG		
GAPDH-F	AGGTCGGAGTGAACGGATTT	62	93
GAPDH-R	GATCATTGATGGCGACAACA		
